# 7′-Phenyl-1′,3′,5′,6′,7′,7a’-hexa­hydro­dipiro[acenaphthyl­ene-1,5′-pyrrolo­[1,2-*c*]thia­zole-6′,2′′-indane]-2,1′′(1*H*)-dione

**DOI:** 10.1107/S1600536812013293

**Published:** 2012-03-31

**Authors:** Ang Chee Wei, Mohamed Ashraf Ali, Tan Soo Choon, Suhana Arshad, Ibrahim Abdul Razak

**Affiliations:** a Institute for Research in Molecular Medicine, Universiti Sains Malaysia, Minden 11800, Penang, Malaysia; b School of Physics, Universiti Sains Malaysia, 11800 USM, Penang, Malaysia

## Abstract

In the title compound, C_31_H_23_NO_2_S, the pyrrolidine ring adopts an envelope conformation (with the spiro C atom as the flap), while the thia­zolidine ring and the two cyclo­pentane rings adopt twisted conformations. The mean plane through the hexa­hydro­pyrrolo­[1,2-*c*]thia­zole ring [r.m.s deviation = 0.400 (1) Å] forms dihedral angles of 76.83 (4), 80.70 (5) and 79.00 (4)° with the benzene ring and the mean planes of the dihydro­acenaphthyl­ene and the dihydro­indene rings, respectively. In the crystal, mol­ecules are linked by C—H⋯O hydrogen bonds into sheets lying parallel to the *bc* plane. One of the ketone O atoms accepts three such bonds. Weak C—H⋯π inter­actions are also observed.

## Related literature

For related structures, see: Wei *et al.* (2011*a* ▶,*b* ▶, 2012 ▶). For ring conformations, see: Cremer & Pople (1975 ▶). For the stability of the temperature controller used in the data collection, see: Cosier & Glazer (1986 ▶).
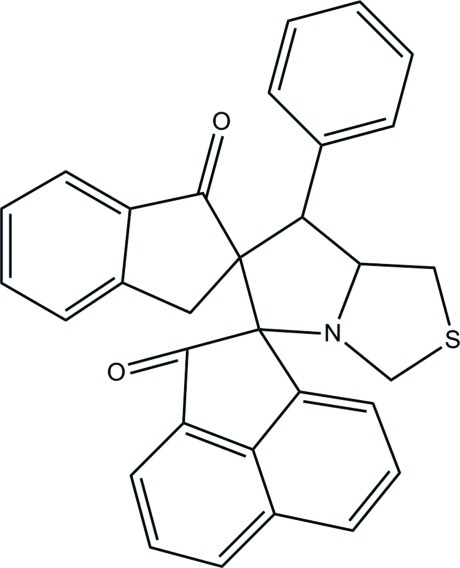


## Experimental

### 

#### Crystal data

C_31_H_23_NO_2_S
*M* *_r_* = 473.56
Monoclinic, 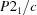

*a* = 8.4054 (1) Å
*b* = 11.3716 (1) Å
*c* = 23.5194 (2) Å
β = 92.259 (1)°
*V* = 2246.30 (4) Å^3^
*Z* = 4
Mo *K*α radiation
μ = 0.18 mm^−1^
*T* = 100 K
0.30 × 0.18 × 0.16 mm

#### Data collection

Bruker SMART APEXII CCD diffractometer
Absorption correction: multi-scan (*SADABS*; Bruker, 2009 ▶) *T*_min_ = 0.949, *T*_max_ = 0.972
39597 measured reflections
10047 independent reflections
7694 reflections with *I* > 2σ(*I*)
*R*_int_ = 0.039

#### Refinement

*R*[*F* ^2^ > 2σ(*F* ^2^)] = 0.051
*wR*(*F* ^2^) = 0.124
*S* = 1.03
10047 reflections
316 parameters
H-atom parameters constrained
Δρ_max_ = 0.58 e Å^−3^
Δρ_min_ = −0.29 e Å^−3^

### 

Data collection: *APEX2* (Bruker, 2009 ▶); cell refinement: *SAINT* (Bruker, 2009 ▶); data reduction: *SAINT*; program(s) used to solve structure: *SHELXTL* (Sheldrick, 2008 ▶); program(s) used to refine structure: *SHELXTL*; molecular graphics: *SHELXTL*; software used to prepare material for publication: *SHELXTL* and *PLATON* (Spek, 2009 ▶).

## Supplementary Material

Crystal structure: contains datablock(s) global, I. DOI: 10.1107/S1600536812013293/hb6700sup1.cif

Structure factors: contains datablock(s) I. DOI: 10.1107/S1600536812013293/hb6700Isup2.hkl

Additional supplementary materials:  crystallographic information; 3D view; checkCIF report

## Figures and Tables

**Table 1:** Hydrogen-bond geometry (Å, °) *Cg*1 and *Cg*2 are the centroids of the C2–C6/C11 and C15–C20 rings, respectively.

*D*—H⋯*A*	*D*—H	H⋯*A*	*D*⋯*A*	*D*—H⋯*A*
C4—H4*A*⋯O1^i^	0.95	2.58	3.2598 (14)	129
C23—H23*A*⋯O1^ii^	1.00	2.46	3.4180 (14)	160
C31—H31*A*⋯O1^ii^	0.95	2.56	3.4434 (14)	155
C7—H7*A*⋯O2^iii^	0.95	2.54	3.4111 (14)	152
C18—H18*A*⋯*Cg*1^iv^	0.95	2.91	3.5502 (14)	126
C25—H25*A*⋯*Cg*2^v^	0.99	2.68	3.5182 (13)	142

Symmetry codes: (i) 
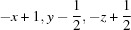
; (ii) 
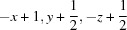
; (iii) 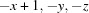; (iv) 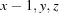; (v) 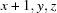.

**Fig. 1.**
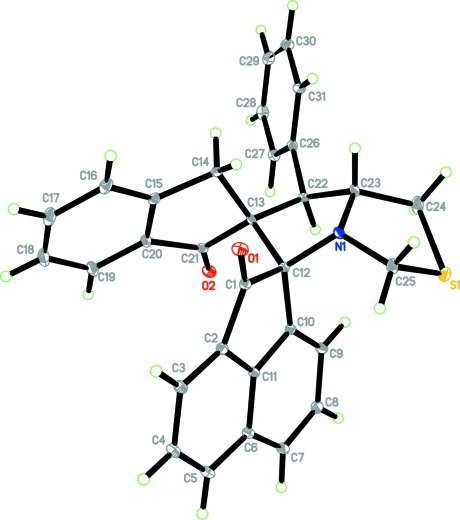
The molecular structure of the title compound, showing 30% probability displacement ellipsoids.

**Fig. 2.**
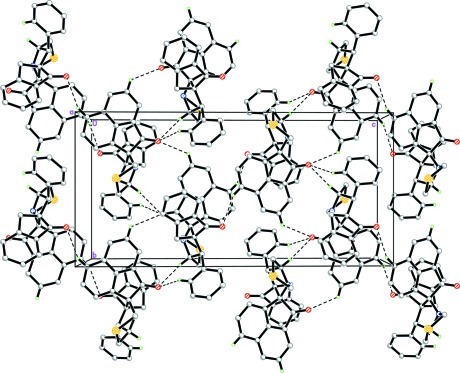
The crystal packing of the title compound. The H atoms not involved in the intermolecular interactions (dashed lines) have been omitted for clarity.

## supplementary crystallographic information

### Comment

As part of our ongoing search to prepare heterocyclic compounds with potential antitubercular activity (Wei *et al.*, 2011*a*,*b*), we have synthesized the title compound as described below.

In the molecular structure (Fig 1), the pyrrolidine ring (N1/C12/C13/C22/C23) is in envelope conformation (Cremer & Pople, 1975) [puckering parameters, Q= 0.4480 (11) Å and φ= 68.75 (14)° with atom C13 at the flap]. Meanwhile, the thiazolidine ring and the two cyclopentane rings (S1/N1/C23–C25, C1/C2/C10–C12 & C13–C15/C20/C21) are twisted about C25–S1 bond [puckering parameters, Q= 0.3450 (11) Å and φ= 339.37 (19)°], C12–C1 bond [puckering parameters, Q= 0.1209 (11) Å and φ= 167.0 (5)°] and C13–S14 bond [puckering parameters, Q= 0.2875 (11) Å and φ= 190.5 (2)°], respectively, adopting half-chair conformation. In addition, the dihedral angles between the mean plane through the hexahydropyrrolo [1,2-*c*]thiazole ring (S1/N1/C12/C13/C22–C25) [r.m.s deviation of 0.400 (1) Å] with the benzene ring (C26–C31) and the mean planes of the dihydroacenaphthylene and the dihydro-indene rings (C1–C10/C12 & C13–C21) are 76.83 (4), 80.70 (5) and 79.00 (4)°, respectively. The bond lengths and angles are within normal ranges and comparable to the related structure (Wei, *et al.*, 2011*b*; Wei, *et al.*, 2012).

The crystal packing is shown in Fig. 2. The molecules are linked into sheets lying parallel to *bc*-plane *via* C7—H7A···O2, C4—H4A···O1, C23—H23A···O1 and C31—H31A···O1 (Table 1) hydrogen bonds. The crystal structure also features C18—H18A···*Cg*1 and C25—H25A···*Cg*2 (Table 1) interactions (*Cg*1 and *Cg*2 are the centroids of the C2–C6/C11 and C15–C20 rings, respectively).

### Experimental

A mixture of (*E*)-(2-benzylidene)-2,3-dihydro-1*H*-indene-1-one (0.001 mol), acenaphthenequinone (0.001 mol) and thiazolidine-4-carboxylic acid (0.002 mol) (1:1:2) were dissolved in methanol (10 ml) and refluxed for 4 h. After completion of the reaction as evident from TLC, the excess solvent was evaporated slowly and the product was separated and recrystallized from methanol to reveal the title compound as yellow crystals.

### Refinement

All H atoms were positioned geometrically (C–H = 0.95 and 1.00 Å) and refined using a riding model with *U*_iso_(H) = 1.2 *U*_eq_(C).

### Crystal data

**Table:**

C_31_H_23_NO_2_S	*F*(000) = 992
*M**_r_* = 473.56	*D*_x_ = 1.400 Mg m^−^^3^
Monoclinic, *P*2_1_/*c*	Mo *K*α radiation, λ = 0.71073 Å
Hall symbol: -P 2ybc	Cell parameters from 9949 reflections
*a* = 8.4054 (1) Å	θ = 2.5–35.2°
*b* = 11.3716 (1) Å	µ = 0.18 mm^−^^1^
*c* = 23.5194 (2) Å	*T* = 100 K
β = 92.259 (1)°	Block, yellow
*V* = 2246.30 (4) Å^3^	0.30 × 0.18 × 0.16 mm
*Z* = 4	

### Data collection

**Table:**

Bruker SMART APEXII CCD diffractometer	10047 independent reflections
Radiation source: fine-focus sealed tube	7694 reflections with *I* > 2σ(*I*)
Graphite monochromator	*R*_int_ = 0.039
φ and ω scans	θ_max_ = 35.3°, θ_min_ = 2.0°
Absorption correction: multi-scan (*SADABS*; Bruker, 2009)	*h* = −13→13
*T*_min_ = 0.949, *T*_max_ = 0.972	*k* = −18→14
39597 measured reflections	*l* = −38→28

### Refinement

**Table:**

Refinement on *F*^2^	Primary atom site location: structure-invariant direct methods
Least-squares matrix: full	Secondary atom site location: difference Fourier map
*R*[*F*^2^ > 2σ(*F*^2^)] = 0.051	Hydrogen site location: inferred from neighbouring sites
*wR*(*F*^2^) = 0.124	H-atom parameters constrained
*S* = 1.03	*w* = 1/[σ^2^(*F*_o_^2^) + (0.0523*P*)^2^ + 0.9508*P*] where *P* = (*F*_o_^2^ + 2*F*_c_^2^)/3
10047 reflections	(Δ/σ)_max_ = 0.001
316 parameters	Δρ_max_ = 0.58 e Å^−^^3^
0 restraints	Δρ_min_ = −0.29 e Å^−^^3^

### Special details

**Table:**

Experimental. The crystal was placed in the cold stream of an Oxford Cryosystems Cobra open-flow nitrogen cryostat (Cosier & Glazer, 1986) operating at 100.0 (1) K.
Geometry. All e.s.d.'s (except the e.s.d. in the dihedral angle between two l.s. planes) are estimated using the full covariance matrix. The cell e.s.d.'s are taken into account individually in the estimation of e.s.d.'s in distances, angles and torsion angles; correlations between e.s.d.'s in cell parameters are only used when they are defined by crystal symmetry. An approximate (isotropic) treatment of cell e.s.d.'s is used for estimating e.s.d.'s involving l.s. planes.
Refinement. Refinement of *F*^2^ against ALL reflections. The weighted *R*-factor *wR* and goodness of fit *S* are based on *F*^2^, conventional *R*-factors *R* are based on *F*, with *F* set to zero for negative *F*^2^. The threshold expression of *F*^2^ > σ(*F*^2^) is used only for calculating *R*-factors(gt) *etc*. and is not relevant to the choice of reflections for refinement. *R*-factors based on *F*^2^ are statistically about twice as large as those based on *F*, and *R*- factors based on ALL data will be even larger.

### Fractional atomic coordinates and isotropic or equivalent isotropic displacement parameters (Å^2^)

**Table:**

	*x*	*y*	*z*	*U*_iso_*/*U*_eq_	
S1	0.86000 (4)	0.42837 (3)	0.121701 (14)	0.01913 (7)	
O1	0.48435 (10)	0.17004 (8)	0.24386 (3)	0.01480 (15)	
O2	0.24182 (10)	0.24847 (8)	0.04159 (3)	0.01487 (16)	
N1	0.60468 (11)	0.35224 (8)	0.17368 (4)	0.01107 (16)	
C1	0.49807 (12)	0.15401 (9)	0.19309 (4)	0.01060 (17)	
C2	0.51959 (12)	0.04064 (9)	0.16415 (4)	0.01095 (18)	
C3	0.50561 (13)	−0.07416 (10)	0.18207 (5)	0.01361 (19)	
H3A	0.4668	−0.0923	0.2184	0.016*	
C4	0.55106 (14)	−0.16396 (10)	0.14435 (5)	0.0159 (2)	
H4A	0.5401	−0.2437	0.1556	0.019*	
C5	0.61100 (14)	−0.13945 (10)	0.09164 (5)	0.0153 (2)	
H5A	0.6423	−0.2022	0.0679	0.018*	
C6	0.62624 (13)	−0.02169 (10)	0.07274 (5)	0.01244 (18)	
C7	0.68707 (13)	0.01653 (10)	0.02044 (5)	0.0147 (2)	
H7A	0.7235	−0.0392	−0.0062	0.018*	
C8	0.69293 (14)	0.13492 (10)	0.00849 (5)	0.0147 (2)	
H8A	0.7345	0.1592	−0.0266	0.018*	
C9	0.63933 (13)	0.22232 (10)	0.04655 (5)	0.01278 (18)	
H9A	0.6441	0.3032	0.0367	0.015*	
C10	0.58047 (12)	0.18838 (9)	0.09778 (4)	0.01008 (17)	
C11	0.57568 (12)	0.06664 (9)	0.10990 (4)	0.01046 (17)	
C12	0.51304 (12)	0.25553 (9)	0.14809 (4)	0.00966 (17)	
C13	0.34938 (12)	0.32033 (9)	0.13396 (4)	0.00936 (17)	
C14	0.25058 (12)	0.32869 (10)	0.18797 (4)	0.01128 (18)	
H14A	0.3205	0.3309	0.2228	0.014*	
H14B	0.1818	0.3994	0.1869	0.014*	
C15	0.15252 (12)	0.21773 (10)	0.18530 (5)	0.01242 (18)	
C16	0.07876 (14)	0.15873 (11)	0.22895 (5)	0.0173 (2)	
H16A	0.0894	0.1859	0.2671	0.021*	
C17	−0.01086 (15)	0.05901 (12)	0.21539 (6)	0.0217 (2)	
H17A	−0.0608	0.0174	0.2448	0.026*	
C18	−0.02897 (15)	0.01885 (12)	0.15930 (6)	0.0221 (2)	
H18A	−0.0930	−0.0483	0.1510	0.027*	
C19	0.04614 (14)	0.07663 (11)	0.11552 (6)	0.0176 (2)	
H19A	0.0346	0.0501	0.0773	0.021*	
C20	0.13885 (13)	0.17474 (10)	0.12972 (5)	0.01284 (18)	
C21	0.24112 (12)	0.24687 (9)	0.09327 (4)	0.01074 (17)	
C22	0.40993 (12)	0.43806 (9)	0.10995 (4)	0.01032 (17)	
H22A	0.4528	0.4210	0.0717	0.012*	
C23	0.55326 (12)	0.46704 (10)	0.15034 (5)	0.01232 (18)	
H23A	0.5157	0.5170	0.1821	0.015*	
C24	0.69479 (14)	0.52975 (11)	0.12283 (6)	0.0225 (3)	
H24A	0.7256	0.6006	0.1451	0.027*	
H24B	0.6638	0.5545	0.0836	0.027*	
C25	0.77507 (13)	0.34147 (11)	0.17878 (5)	0.0159 (2)	
H25A	0.8066	0.2580	0.1753	0.019*	
H25B	0.8146	0.3710	0.2163	0.019*	
C26	0.28586 (12)	0.53385 (9)	0.10170 (4)	0.01095 (17)	
C27	0.20247 (13)	0.54334 (10)	0.04912 (5)	0.01402 (19)	
H27A	0.2268	0.4909	0.0192	0.017*	
C28	0.08491 (14)	0.62787 (11)	0.03980 (5)	0.0170 (2)	
H28A	0.0287	0.6319	0.0040	0.020*	
C29	0.04933 (14)	0.70648 (11)	0.08275 (5)	0.0176 (2)	
H29A	−0.0311	0.7643	0.0765	0.021*	
C30	0.13264 (14)	0.69968 (10)	0.13495 (5)	0.0163 (2)	
H30A	0.1102	0.7540	0.1642	0.020*	
C31	0.24880 (13)	0.61373 (10)	0.14462 (5)	0.01328 (19)	
H31A	0.3035	0.6092	0.1807	0.016*	

### Atomic displacement parameters (Å^2^)

**Table:**

	*U*^11^	*U*^22^	*U*^33^	*U*^12^	*U*^13^	*U*^23^
S1	0.01378 (12)	0.01626 (14)	0.02775 (15)	−0.00203 (10)	0.00564 (10)	0.00049 (11)
O1	0.0201 (4)	0.0142 (4)	0.0101 (3)	0.0016 (3)	0.0005 (3)	0.0002 (3)
O2	0.0185 (4)	0.0146 (4)	0.0114 (3)	−0.0002 (3)	−0.0016 (3)	−0.0021 (3)
N1	0.0106 (4)	0.0089 (4)	0.0135 (4)	−0.0008 (3)	−0.0017 (3)	−0.0008 (3)
C1	0.0104 (4)	0.0104 (4)	0.0110 (4)	0.0011 (3)	−0.0002 (3)	0.0010 (3)
C2	0.0123 (4)	0.0094 (4)	0.0111 (4)	0.0004 (3)	−0.0002 (3)	0.0004 (3)
C3	0.0159 (4)	0.0113 (5)	0.0136 (4)	−0.0005 (4)	0.0000 (3)	0.0014 (4)
C4	0.0202 (5)	0.0095 (5)	0.0180 (5)	−0.0007 (4)	0.0002 (4)	0.0012 (4)
C5	0.0185 (5)	0.0104 (5)	0.0169 (5)	0.0005 (4)	0.0000 (4)	−0.0019 (4)
C6	0.0142 (4)	0.0098 (4)	0.0133 (4)	0.0003 (4)	0.0004 (3)	−0.0017 (3)
C7	0.0168 (5)	0.0139 (5)	0.0138 (4)	−0.0009 (4)	0.0031 (4)	−0.0041 (4)
C8	0.0170 (5)	0.0149 (5)	0.0123 (4)	−0.0019 (4)	0.0041 (4)	−0.0016 (4)
C9	0.0154 (4)	0.0109 (4)	0.0123 (4)	−0.0006 (4)	0.0031 (3)	−0.0001 (3)
C10	0.0106 (4)	0.0090 (4)	0.0107 (4)	0.0006 (3)	0.0009 (3)	−0.0001 (3)
C11	0.0112 (4)	0.0087 (4)	0.0114 (4)	0.0000 (3)	0.0004 (3)	−0.0001 (3)
C12	0.0109 (4)	0.0084 (4)	0.0097 (4)	−0.0002 (3)	0.0004 (3)	−0.0001 (3)
C13	0.0098 (4)	0.0088 (4)	0.0095 (4)	−0.0005 (3)	0.0000 (3)	−0.0009 (3)
C14	0.0118 (4)	0.0108 (4)	0.0113 (4)	0.0008 (3)	0.0020 (3)	−0.0005 (3)
C15	0.0099 (4)	0.0128 (5)	0.0146 (4)	0.0021 (4)	0.0015 (3)	0.0024 (4)
C16	0.0146 (5)	0.0194 (6)	0.0183 (5)	0.0016 (4)	0.0043 (4)	0.0062 (4)
C17	0.0147 (5)	0.0195 (6)	0.0310 (6)	−0.0005 (4)	0.0044 (4)	0.0122 (5)
C18	0.0149 (5)	0.0151 (5)	0.0362 (7)	−0.0035 (4)	−0.0012 (5)	0.0064 (5)
C19	0.0148 (5)	0.0127 (5)	0.0251 (6)	−0.0027 (4)	−0.0031 (4)	0.0012 (4)
C20	0.0116 (4)	0.0112 (5)	0.0156 (5)	−0.0006 (4)	−0.0007 (3)	0.0012 (4)
C21	0.0109 (4)	0.0083 (4)	0.0128 (4)	0.0013 (3)	−0.0014 (3)	−0.0006 (3)
C22	0.0116 (4)	0.0086 (4)	0.0108 (4)	0.0000 (3)	0.0004 (3)	−0.0005 (3)
C23	0.0116 (4)	0.0083 (4)	0.0169 (5)	0.0001 (3)	−0.0016 (3)	−0.0017 (4)
C24	0.0140 (5)	0.0142 (5)	0.0390 (7)	−0.0026 (4)	−0.0021 (5)	0.0093 (5)
C25	0.0122 (4)	0.0133 (5)	0.0219 (5)	−0.0007 (4)	−0.0038 (4)	0.0025 (4)
C26	0.0111 (4)	0.0092 (4)	0.0125 (4)	−0.0005 (3)	0.0003 (3)	0.0011 (3)
C27	0.0156 (4)	0.0132 (5)	0.0132 (4)	−0.0006 (4)	−0.0007 (3)	0.0019 (4)
C28	0.0153 (5)	0.0166 (5)	0.0188 (5)	−0.0007 (4)	−0.0029 (4)	0.0065 (4)
C29	0.0139 (5)	0.0131 (5)	0.0260 (6)	0.0023 (4)	0.0018 (4)	0.0068 (4)
C30	0.0164 (5)	0.0114 (5)	0.0215 (5)	0.0024 (4)	0.0042 (4)	0.0004 (4)
C31	0.0147 (4)	0.0105 (5)	0.0146 (4)	0.0013 (4)	0.0007 (3)	−0.0005 (4)

### Geometric parameters (Å, º)

**Table:**

S1—C24	1.8058 (13)	C14—H14B	0.9900
S1—C25	1.8340 (12)	C15—C16	1.3923 (15)
O1—C1	1.2178 (12)	C15—C20	1.3961 (16)
O2—C21	1.2159 (13)	C16—C17	1.3914 (19)
N1—C25	1.4377 (14)	C16—H16A	0.9500
N1—C12	1.4589 (14)	C17—C18	1.398 (2)
N1—C23	1.4743 (14)	C17—H17A	0.9500
C1—C2	1.4724 (15)	C18—C19	1.3936 (18)
C1—C12	1.5745 (15)	C18—H18A	0.9500
C2—C3	1.3782 (15)	C19—C20	1.3939 (16)
C2—C11	1.4090 (14)	C19—H19A	0.9500
C3—C4	1.4151 (16)	C20—C21	1.4849 (15)
C3—H3A	0.9500	C22—C26	1.5152 (15)
C4—C5	1.3849 (16)	C22—C23	1.5403 (15)
C4—H4A	0.9500	C22—H22A	1.0000
C5—C6	1.4184 (16)	C23—C24	1.5502 (16)
C5—H5A	0.9500	C23—H23A	1.0000
C6—C11	1.4080 (15)	C24—H24A	0.9900
C6—C7	1.4187 (15)	C24—H24B	0.9900
C7—C8	1.3766 (17)	C25—H25A	0.9900
C7—H7A	0.9500	C25—H25B	0.9900
C8—C9	1.4227 (15)	C26—C27	1.4014 (15)
C8—H8A	0.9500	C26—C31	1.4021 (15)
C9—C10	1.3757 (14)	C27—C28	1.3898 (16)
C9—H9A	0.9500	C27—H27A	0.9500
C10—C11	1.4142 (15)	C28—C29	1.3903 (18)
C10—C12	1.5355 (14)	C28—H28A	0.9500
C12—C13	1.5842 (14)	C29—C30	1.3914 (18)
C13—C21	1.5405 (15)	C29—H29A	0.9500
C13—C22	1.5469 (15)	C30—C31	1.3938 (16)
C13—C14	1.5477 (14)	C30—H30A	0.9500
C14—C15	1.5071 (16)	C31—H31A	0.9500
C14—H14A	0.9900		
			
C24—S1—C25	90.66 (6)	C17—C16—H16A	120.8
C25—N1—C12	118.57 (9)	C15—C16—H16A	120.8
C25—N1—C23	112.52 (9)	C16—C17—C18	121.35 (11)
C12—N1—C23	111.82 (8)	C16—C17—H17A	119.3
O1—C1—C2	127.04 (10)	C18—C17—H17A	119.3
O1—C1—C12	124.22 (10)	C19—C18—C17	120.49 (12)
C2—C1—C12	108.47 (8)	C19—C18—H18A	119.8
C3—C2—C11	120.75 (10)	C17—C18—H18A	119.8
C3—C2—C1	132.42 (10)	C18—C19—C20	117.75 (12)
C11—C2—C1	106.65 (9)	C18—C19—H19A	121.1
C2—C3—C4	117.61 (10)	C20—C19—H19A	121.1
C2—C3—H3A	121.2	C19—C20—C15	121.95 (10)
C4—C3—H3A	121.2	C19—C20—C21	129.39 (11)
C5—C4—C3	122.18 (11)	C15—C20—C21	108.61 (9)
C5—C4—H4A	118.9	O2—C21—C20	127.67 (10)
C3—C4—H4A	118.9	O2—C21—C13	125.94 (10)
C4—C5—C6	120.74 (10)	C20—C21—C13	106.38 (9)
C4—C5—H5A	119.6	C26—C22—C23	116.28 (9)
C6—C5—H5A	119.6	C26—C22—C13	115.74 (8)
C11—C6—C5	116.48 (10)	C23—C22—C13	102.74 (8)
C11—C6—C7	116.58 (10)	C26—C22—H22A	107.2
C5—C6—C7	126.94 (10)	C23—C22—H22A	107.2
C8—C7—C6	119.58 (10)	C13—C22—H22A	107.2
C8—C7—H7A	120.2	N1—C23—C22	104.53 (8)
C6—C7—H7A	120.2	N1—C23—C24	110.13 (9)
C7—C8—C9	122.68 (10)	C22—C23—C24	115.81 (10)
C7—C8—H8A	118.7	N1—C23—H23A	108.7
C9—C8—H8A	118.7	C22—C23—H23A	108.7
C10—C9—C8	119.27 (10)	C24—C23—H23A	108.7
C10—C9—H9A	120.4	C23—C24—S1	108.40 (8)
C8—C9—H9A	120.4	C23—C24—H24A	110.0
C9—C10—C11	117.72 (9)	S1—C24—H24A	110.0
C9—C10—C12	133.78 (10)	C23—C24—H24B	110.0
C11—C10—C12	108.49 (8)	S1—C24—H24B	110.0
C6—C11—C2	122.16 (10)	H24A—C24—H24B	108.4
C6—C11—C10	124.16 (9)	N1—C25—S1	107.95 (8)
C2—C11—C10	113.65 (9)	N1—C25—H25A	110.1
N1—C12—C10	119.20 (8)	S1—C25—H25A	110.1
N1—C12—C1	109.29 (8)	N1—C25—H25B	110.1
C10—C12—C1	101.25 (8)	S1—C25—H25B	110.1
N1—C12—C13	100.13 (8)	H25A—C25—H25B	108.4
C10—C12—C13	114.42 (8)	C27—C26—C31	117.89 (10)
C1—C12—C13	112.98 (8)	C27—C26—C22	119.08 (9)
C21—C13—C22	115.93 (8)	C31—C26—C22	123.02 (10)
C21—C13—C14	102.72 (8)	C28—C27—C26	121.35 (11)
C22—C13—C14	116.20 (8)	C28—C27—H27A	119.3
C21—C13—C12	111.40 (8)	C26—C27—H27A	119.3
C22—C13—C12	100.58 (8)	C27—C28—C29	120.14 (11)
C14—C13—C12	110.24 (8)	C27—C28—H28A	119.9
C15—C14—C13	102.98 (8)	C29—C28—H28A	119.9
C15—C14—H14A	111.2	C28—C29—C30	119.35 (11)
C13—C14—H14A	111.2	C28—C29—H29A	120.3
C15—C14—H14B	111.2	C30—C29—H29A	120.3
C13—C14—H14B	111.2	C29—C30—C31	120.52 (11)
H14A—C14—H14B	109.1	C29—C30—H30A	119.7
C16—C15—C20	119.94 (11)	C31—C30—H30A	119.7
C16—C15—C14	129.15 (11)	C30—C31—C26	120.73 (11)
C20—C15—C14	110.90 (9)	C30—C31—H31A	119.6
C17—C16—C15	118.45 (12)	C26—C31—H31A	119.6
			
O1—C1—C2—C3	11.8 (2)	C12—C13—C14—C15	−90.66 (10)
C12—C1—C2—C3	−173.99 (11)	C13—C14—C15—C16	158.41 (11)
O1—C1—C2—C11	−163.15 (11)	C13—C14—C15—C20	−22.15 (11)
C12—C1—C2—C11	11.09 (11)	C20—C15—C16—C17	−1.61 (17)
C11—C2—C3—C4	0.66 (16)	C14—C15—C16—C17	177.78 (11)
C1—C2—C3—C4	−173.68 (11)	C15—C16—C17—C18	−0.76 (18)
C2—C3—C4—C5	1.38 (17)	C16—C17—C18—C19	1.58 (19)
C3—C4—C5—C6	−1.31 (18)	C17—C18—C19—C20	0.02 (18)
C4—C5—C6—C11	−0.79 (16)	C18—C19—C20—C15	−2.44 (17)
C4—C5—C6—C7	179.17 (11)	C18—C19—C20—C21	174.76 (11)
C11—C6—C7—C8	−0.39 (16)	C16—C15—C20—C19	3.28 (17)
C5—C6—C7—C8	179.66 (12)	C14—C15—C20—C19	−176.22 (10)
C6—C7—C8—C9	−0.32 (18)	C16—C15—C20—C21	−174.43 (10)
C7—C8—C9—C10	0.68 (17)	C14—C15—C20—C21	6.07 (12)
C8—C9—C10—C11	−0.29 (16)	C19—C20—C21—O2	15.01 (19)
C8—C9—C10—C12	−179.68 (11)	C15—C20—C21—O2	−167.50 (11)
C5—C6—C11—C2	2.84 (16)	C19—C20—C21—C13	−164.61 (11)
C7—C6—C11—C2	−177.12 (10)	C15—C20—C21—C13	12.88 (12)
C5—C6—C11—C10	−179.25 (10)	C22—C13—C21—O2	26.92 (15)
C7—C6—C11—C10	0.79 (16)	C14—C13—C21—O2	154.73 (11)
C3—C2—C11—C6	−2.84 (16)	C12—C13—C21—O2	−87.27 (13)
C1—C2—C11—C6	172.80 (10)	C22—C13—C21—C20	−153.45 (9)
C3—C2—C11—C10	179.05 (10)	C14—C13—C21—C20	−25.64 (10)
C1—C2—C11—C10	−5.31 (12)	C12—C13—C21—C20	92.35 (10)
C9—C10—C11—C6	−0.45 (16)	C21—C13—C22—C26	70.82 (11)
C12—C10—C11—C6	179.09 (10)	C14—C13—C22—C26	−49.99 (12)
C9—C10—C11—C2	177.62 (10)	C12—C13—C22—C26	−168.95 (8)
C12—C10—C11—C2	−2.84 (12)	C21—C13—C22—C23	−161.38 (8)
C25—N1—C12—C10	−37.51 (14)	C14—C13—C22—C23	77.81 (10)
C23—N1—C12—C10	95.94 (11)	C12—C13—C22—C23	−41.15 (9)
C25—N1—C12—C1	78.11 (11)	C25—N1—C23—C22	140.45 (9)
C23—N1—C12—C1	−148.44 (8)	C12—N1—C23—C22	4.10 (11)
C25—N1—C12—C13	−163.03 (9)	C25—N1—C23—C24	15.42 (13)
C23—N1—C12—C13	−29.58 (10)	C12—N1—C23—C24	−120.94 (10)
C9—C10—C12—N1	−51.77 (16)	C26—C22—C23—N1	151.58 (9)
C11—C10—C12—N1	128.81 (10)	C13—C22—C23—N1	24.12 (10)
C9—C10—C12—C1	−171.57 (12)	C26—C22—C23—C24	−87.07 (12)
C11—C10—C12—C1	9.00 (10)	C13—C22—C23—C24	145.48 (9)
C9—C10—C12—C13	66.60 (15)	N1—C23—C24—S1	7.73 (12)
C11—C10—C12—C13	−112.83 (10)	C22—C23—C24—S1	−110.57 (10)
O1—C1—C12—N1	35.65 (13)	C25—S1—C24—C23	−21.26 (10)
C2—C1—C12—N1	−138.80 (9)	C12—N1—C25—S1	101.83 (9)
O1—C1—C12—C10	162.27 (10)	C23—N1—C25—S1	−31.32 (11)
C2—C1—C12—C10	−12.17 (10)	C24—S1—C25—N1	30.36 (9)
O1—C1—C12—C13	−74.89 (13)	C23—C22—C26—C27	147.99 (10)
C2—C1—C12—C13	110.66 (9)	C13—C22—C26—C27	−91.27 (12)
N1—C12—C13—C21	166.23 (8)	C23—C22—C26—C31	−32.59 (14)
C10—C12—C13—C21	37.52 (11)	C13—C22—C26—C31	88.14 (12)
C1—C12—C13—C21	−77.65 (10)	C31—C26—C27—C28	−0.97 (16)
N1—C12—C13—C22	42.79 (9)	C22—C26—C27—C28	178.47 (10)
C10—C12—C13—C22	−85.92 (10)	C26—C27—C28—C29	0.96 (17)
C1—C12—C13—C22	158.92 (8)	C27—C28—C29—C30	0.09 (17)
N1—C12—C13—C14	−80.40 (9)	C28—C29—C30—C31	−1.11 (17)
C10—C12—C13—C14	150.88 (9)	C29—C30—C31—C26	1.10 (17)
C1—C12—C13—C14	35.72 (11)	C27—C26—C31—C30	−0.06 (16)
C21—C13—C14—C15	28.15 (10)	C22—C26—C31—C30	−179.48 (10)
C22—C13—C14—C15	155.79 (9)		

### Hydrogen-bond geometry (Å, º)

Cg1 and Cg2 are the centroids of the C2–C6/C11 and C15–C20 rings, respectively.

**Table:**

*D*—H···*A*	*D*—H	H···*A*	*D*···*A*	*D*—H···*A*
C4—H4*A*···O1^i^	0.95	2.58	3.2598 (14)	129
C23—H23*A*···O1^ii^	1.00	2.46	3.4180 (14)	160
C31—H31*A*···O1^ii^	0.95	2.56	3.4434 (14)	155
C7—H7*A*···O2^iii^	0.95	2.54	3.4111 (14)	152
C18—H18*A*···*Cg*1^iv^	0.95	2.91	3.5502 (14)	126
C25—H25*A*···*Cg*2^v^	0.99	2.68	3.5182 (13)	142

Symmetry codes: (i) −*x*+1, *y*−1/2, −*z*+1/2; (ii) −*x*+1, *y*+1/2, −*z*+1/2; (iii) −*x*+1, −*y*, −*z*; (iv) *x*−1, *y*, *z*; (v) *x*+1, *y*, *z*.
